# Positive and negative adjustment in couples undergoing infertility treatment: The impact of support exchange

**DOI:** 10.1371/journal.pone.0200124

**Published:** 2018-06-28

**Authors:** Aleksandra Kroemeke, Ewelina Kubicka

**Affiliations:** Department of Psychology, SWPS University of Social Sciences and Humanities, Warsaw, Poland; Monash University, Institute of Cognitive and Clinical Neurosciences, AUSTRALIA

## Abstract

**Background:**

Perceived social support relates to infertility-related distress in couples undergoing assisted reproductive technology (ART) treatment. Studies examining the effect of other support types on both positive and negative adjustment among infertile couples are scarce or non-existent. Therefore, this study investigated the effects of support receipt, provision, invisibility (the discrepancy between one partner’s received and the other partner’s provided support), and equity (the discrepancy between each partner’s received and provided support) on the positive (life purpose) and negative (depressive symptoms) indices of well-being in couples undergoing ART treatment.

**Methods:**

Depressive symptoms (CES-D), life purpose (PIL), and social support (BSSS) were assessed among 31 married couples (mean age 32.67 years) undergoing ART treatment. Data were analyzed by applying the Actor-Partner-Interdependence Model (APIM) using multilevel modeling.

**Findings:**

Both receiving and providing support had beneficial effects in women and men. However, sub-analysis showed differences according to gender and the support exchange effects. Women reported higher depression and lower life purpose but benefited more from support, and their well-being was more dependent on their own perception of support provision and receipt. Men demonstrated higher adjustment to infertility but benefited less from support, and their well-being was mostly correlated with supportive behaviors of their wives.

**Discussion:**

Adjustment mechanisms of women and men undergoing ART treatment vary considerably; thus, gender should be taken into consideration in interventions. Future studies should focus on costs/benefits and gender differences of visible and invisible support in infertility settings.

## Introduction

Infertility and assisted reproductive technology (ART) treatment are highly stressful events, which may significantly affect the well-being of spouses in different life domains [1–3]. Studies on gender differences in psychological adjustment to infertility found that women in particular are at high risk for developing psychopathologies in these settings [4]. However, conclusions about the general well-being of infertile couples based on these findings are limited, as most studies focus on negative indices of adaptation, and thus tend to be pathogenically oriented.

Social support seems to be particularly important in the context of infertility. As infertility and its treatment is a couple-level stressor, it involves both spouses, who constitute the primary source of support for each other. Previous studies have found that perceived social support was negatively related to distress, depression, or anxiety in both cross-sectional [5–8] and longitudinal [9] studies on infertile individuals. Dyadic studies have revealed a significant actor effect of perceived social support on distress in both wives and husbands, but a significant partner effect only for husbands [10]. The perception of social support in infertile couples has been extensively studied. Regardless, earlier research focused on perceived support, whereas received support was largely unaddressed. Meanwhile, social exchange in a dyad involves support provision and receipt.

Despite a notably smaller number of studies focusing on provided support, their results suggest its positive relation with couple well-being [11–13], and claim that it may surpass received support in beneficial effects [11], which is compatible with the hypothesis of esteem enhancement [14,15]. In contrast, received support has positive effects [11,16], no effect [17,18], or even adverse effects [19] on well-being. On the one hand, these mixed effects are consistent with equity theory, according to which receiving support may result in distress and guilt when it violates the reciprocity principle of a social exchange, that is, when more is received than given [20]. On the other hand, according to social exchange theory, receiving support should be connected to higher well-being, as people strive for maximization of their profits and minimization of their losses [21]. The discrepancy between provided and received social support is also the basis for the concept of invisible support. In addition to the benefits of being supported, awareness of receiving support may entail an emotional cost, hence, the most effective support is unnoticed by the recipient [22]. Several studies have supported that assumption [23–25], although outside the context of infertility.

### The present study

This study aimed at examining actor and partner effects of provided, received, and invisible support on positive (life purpose) and negative (depressive symptoms) adjustment indices in couples undergoing ART treatment. As proposed by social exchange theory [21], the enhanced esteem hypothesis [14,15], and the model of invisible support [22], it was hypothesized that both spouses would benefit from support receipt, provision, and invisibility (i.e., the discrepancy between received and provided support, indicating that support provided by one partner is greater than support receipt reported by the other partner) by reporting lower depressive symptoms, and higher life purpose. To the best of our knowledge, this study has been the first to address support receipt, provision, and invisibility, and their effects on both well-being indices in infertile couples.

Furthermore, to better examine the social exchange between infertile couples and derive conclusions about the superiority of social exchange theory or equity theory, support equity—the discrepancy between one partner’s support receipt and provision—was tested. Beneficial effects of support equity on adjustment would support social exchange theory. Analysis including the discrepancy in support perception between partners may advance our understanding of benefits of spousal support when coping with infertility. To the best of our knowledge, this study has been the first to address this issue. As for support equity, no hypotheses have been formulated due to the exploratory nature of these analyses.

Finally, gender differences in all actor and partner effects were tested, as previous studies have found differences in the adjustment [4] and social support effects [10] among infertile partners or community couples [26]. Based on earlier findings, it was hypothesized that women would be more depressed, and have lower life purpose, but benefit more from support receipt, provision, and invisible support.

## Methods

### Participants and procedure

The sample included 31 married couples (*N* = 62 participants) undergoing ART treatment in Poland. Mean age of the participants was 31.97±2.98 years for women and 33.37±2.12 years for men (range: 27–38). The mean length of marriage was 6.55±1.99 years (range: 4–14). The clear majority of respondents had higher education (women: 97%, men: 90%) and average socioeconomic status (93%). Duration of infertility was 4.16±2.03 years on average (1.1–9 years), currently treated by monitoring ovulation (19.4%), intrauterine insemination (54.8%), or *in vitro* fertilization (25.8%).

The Ethics Committee of the SWPS University of Social Sciences and Humanities approved the study protocol. Recruitment took place before a standard control visit at a public infertility clinic. In total, 190 patients (95 couples) gave informed consent to participate, and received envelopes with information about the study and questionnaires, as well as pre-addressed stamped return envelopes. The participants were instructed to complete the questionnaires independently. The dropout rate was 67%.

### Measures

#### Depressive symptoms

Depressive symptoms were measured using the Centre for Epidemiological Studies Depression Scale (CES-D) [27,28], which assessed the severity of each symptom on a 4-point scale, from 0 (rarely or never) to 3 (often). A higher result indicated a greater number of depressive symptoms. Cronbach’s α in the current study was .90 and .85 for women and men, respectively (.93 in the whole sample).

#### Life purpose

Life purpose was measured using the abbreviated (6-item) version of the Purpose in Life Test (PIL) [29,30]. In the Polish population, the abbreviated 6-item version has better psychometric properties than the 4-item version proposed by Schulenberg et al. [31]. All statements were assessed on a 7-point scale with different anchoring points for each item. A higher result indicated a greater sense of life purpose. Cronbach’s α in the current study was .85 and .89 for women and men, respectively (.89 in the whole sample).

#### Social support

Received and provided social support was measured using the Berlin Social Support Scales (BSSS) [32]. Participants assessed support from/to spouse. All statements were assessed on a 4-point scale, from 1 (*strongly disagree*) to 4 (*strongly agree*), and averaged. A higher result indicated a greater level of support. In the current study, Cronbach’s α of support receipt was .85 and .89 for women and men, respectively (.86 in the whole sample), whereas Cronbach’s α of support provision was .77 and .83 for women and men, respectively (.80 in the whole sample).

### Data analyses

Multilevel modeling (MLM) with a pairwise dataset was performed using SPSS version 23 to examine the actor and partner effects of received and provided social support, as well as discrepancy indicators of individual spousal adaptation (depressive symptoms and life purpose). MLM is considered one of the best methods to examine effects in the Actor-Partner-Interdependence Model (APIM) [33]. The interaction models (examination of gender differences in actor and partner effects) and two-intercept models (examination of actor and partner effects for wives and husbands, respectively), separately for support receipt, support provision, and two support discrepancy indices as predictors of depressive symptoms and life purpose, were estimated. Discrepancy scores for the actor effects were calculated by subtracting (i) received support reported by an actor from the provided support reported by a partner (invisible support), and (ii) provided support reported by an actor from the received support reported by an actor (support equity). An identical formula was used for the partner effects. Positive values indicated (i) occurrence of invisible support (partner provided > actor received), and (ii) dominance of support receipt (actor received > actor provided). In addition, the main effects of infertility duration and gender were examined (other controlled variables, i.e., age, education, length of marriage, socioeconomic status, and type of treatment were not significantly connected with the adjustment indices, so they were not included in the models). Predictors were grand mean centered to avoid multicollinearity. There were no missing data. The response rate was low, resulting in a relatively small sample size; however, studies indicated that a sample size larger than 25 dyads provided adequate power in the test of nonindependence [34] and that unbiased estimates of fixed effects parameters might be obtained for a small sample size (e.g., 31 dyads) [35–37]. The effect size *r* for each *t* was computed with the following equation: sqrt[*t*^2^/(*t*^2^+*df*)]. Pseudo *R*^2^, defined as 1− (estimates of the variance and covariance of the full model/estimates of the variance and covariance from the null model), was calculated to determine the estimate of variance explained by the predictors, separately for the wives and the husbands [34]. Goodness of fit for the models was based on the –2 Restricted log likelihood ratio, the Akaike Information Criterion (AIC), and the Bayesian Information Criterion (BIC). The model with the lower AIC, BIC, and –2 Restricted log likelihood values indicated better fit [34].

## Results

Descriptive statistics are presented in Table 1. Significant gender differences in adjustment indices were noted, in favor of men. No significant differences between women and men in the support types were found. In addition, the received and provided support in the entire sample was at a similar level (*t*_61_ = 1.435, *p* = 0.156, *M*_rec_ = 3.45±0.04, *M*_prov_ = 3.38±0.05). Partial Pearson correlations between actor and partner variables (Table 2), controlled for gender, indicated a significant association between actor depression and partner life purpose, and support receipt, equity, and visibility. Actor life purpose was significantly related to partner life purpose and invisible support. All coefficients indicated a low-to-moderate effect.

**Table 1:** Descriptive statistics and gender comparisons (*N* = 31 couples).

	Women		Men		*t*_60_
	*M*	*SD*	*M*	*SD*	
Depressive symptoms	1.36	.43	.68	.34	6.84***
Life purpose	5.16	.68	5.92	.70	-4.38***
Received support	3.33	.40	3.43	.47	-.94
Provided support	3.38	.32	3.52	.35	-1.58
Invisible support	-.06	.46	.18	.50	-1.94
Equity support	-.05	.33	-.08	.38	.36

***p < .001.

**Table 2:** Partial correlation, controlled for gender, between the study variables (*N* = 62 participants).

		Partner					
		1.	2.	3.	4.	5.	6.
Actor	1. Depressive symptoms	.24^	-.26*	-.32*	-.09	.31*	-.31*
	2. Life purpose		.53***	.19	.10	-.35**	.13
	3. Received support			-.11	.23	-.74***	-.35**
	4. Provided support				-.03	-.56***	.31*
	5. Invisible support					-.85***	-.37**
	6. Support equity						-.72***

^*p* < .06.

*p < .05.

**p < .01.

***p < .001.

Results of MLM confirmed significant main effects of gender (Tables 3–6). Moreover, a significant main effect of infertility duration was observed. Taken together, female gender and longer duration of infertility were related to poorer adjustment, that is, higher depressive symptoms and lower life purpose.

**Table 3:** Results of MLM of support receipt on depression and life purpose (*N* = 31 couples).

Predictor: received support	Adjustment									
	Depressive symptoms					Life purpose				
	*B*	*SE*	*95% Cl*		*Effect size r*	*B*	*SE*	*95% Cl*		*Effect size r*
			*lower*	*upper*				*lower*	*upper*	
*Fixed effects*										
Intercept	.99***	.03	.92	1.05		5.52***	.09	5.33	5.72	
Actor received support	-.30**	.10	-.50	-.11	.40	.80**	.21	.37	1.22	.53
Partner received support	-.22*	.10	-.42	-.02	.30	.33	.21	-.10	.76	.25
Infertility duration	.10**	.03	.04	.15	.57	.01	.07	-.15	.16	.02
Gender (-1 women, 1 men)	-.33***	.04	-.43	-.24	.82	.36***	.05	.25	.47	.78
Actor received support × gender	.37**	.11	.15	.58	.42	-.03	.23	-.49	.44	.02
Partner received support × gender	-.28*	.11	-.50	-.07	.34	-.37	.23	-.84	.10	.25
*[Co-]variances*										
Covariance (women)	.08**	.02	.05	.15		.30***	.08	.18	.52	
Covariance (men)	.10***	.03	.06	.18		.40***	.11	.23	.70	
Residual variance	.09***	.02	.06	.14		.51***	.14	.19	.74	
*Goodness-of-fit*										
–2 Restricted log likelihood	44.97					111.86				
AIC / BIC	50.97 / 56.99					117.86 / 123.89				
Pseudo *R*^2^—full model (women / men)	61.3% / 27.4%					35.1% / 21.2%				
Pseudo *R*^2^—received support only (women/men)	34.0% / 27.4%					35.1% / 19.5%				

*AIC* Akaike Information Criterion; *BIC* Bayesian Information Criterion. Null model for depression: AIC = 96.14; BIC = 104.64; for life purpose: AIC = 152.50; BIC = 161.01.

*p < .05.

**p < .01.

***p < .001.

**Table 4:** Results of MLM of support provision on depression and life purpose (*N* = 31 couples).

Predictor: provided support	Adjustment									
	Depressive symptoms					Life purpose				
	*B*	*SE*	*95% Cl*		*Effect size r*	*B*	*SE*	*95% Cl*		*Effect size r*
			*lower*	*upper*				*lower*	*upper*	
*Fixed effects*										
Intercept	.98***	.04	.90	1.06		5.49***	.09	5.31	5.67	
Actor provided support	-.67***	.12	-.91	-.43	1.41	1.23***	.22	.78	1.68	.68
Partner provided support	-.13	.12	-.40	.11	.14	.13	.22	-.31	.58	.10
Infertility duration	.01	.02	-.03	.06	.13	-.04	.04	-.14	.05	.17
Gender (-1 women, 1 men)	-.30***	.04	-.38	-.22	.82	.31***	.04	.23	.39	.83
Actor provided support × gender	.31**	.11	.09	.54	.35	.25	.20	-.16	.65	.20
Partner provided support × gender	-.28*	.11	-.51	-.05	.32	-.45*	.19	-.85	-.05	.35
*[Co-]variances*										
Covariance (women)	.08***	.02	.04	.13		.32***	.09	.19	.55	
Covariance (men)	.09***	.02	.05	.16		.21***	.06	.12	.35	
Residual variance	.08***	.02	.06	.12		.69***	.10	.44	.84	
*Goodness-of-fit*										
–2 Restricted log likelihood	41.43					84.52				
AIC / BIC	47.43 / 53.45					90.52 / 96.54				
Pseudo *R*^2^—full model (women / men)	59.4% / 24.1%					29.6% / 58%				
Pseudo *R*^2^—provided support only (women/men)	57.2% / 24.1%					29.5% / 58%				

*AIC* Akaike Information Criterion; *BIC* Bayesian Information Criterion. Null model for depression: AIC = 96.14; BIC = 104.64; for life purpose: AIC = 152.50; BIC = 161.01.

*p < .05.

**p < .01.

***p < .001.

**Table 5:** Results of MLM of invisible support on depression and life purpose (*N* = 31 couples).

Predictor: invisible support	Adjustment									
	Depressive symptoms					Life purpose				
	*B*	*SE*	*95% Cl*		*Effect size r*	*B*	*SE*	*95% Cl*		*Effect size r*
			*lower*	*upper*				*lower*	*upper*	
*Fixed effects*										
Intercept	.97***	.04	.88	1.06		5.49***	.11	5.27	5.71	
Actor invisible support	.11	.17	-.24	.47	.12	-.17	.43	-1.06	.72	.08
Partner invisible support	.28	.18	-.08	.64	.28	-.62	.43	-1.51	.27	.26
Infertility duration	.11***	.02	.06	.16	.67	-.03	.06	-.16	.11	.08
Gender (-1 women, 1 men)	-.32***	.04	-.41	-.23	.82	.33***	.04	.25	.41	.84
Actor invisible support × gender	.08	.16	-.26	.41	.08	.67***	.20	.26	1.08	.42
Partner invisible support × gender	-.34*	.17	-.69	.01	.33	.25	.20	-.15	.65	.17
*[Co-]variances*										
Covariance (women)	.07**	.02	.04	.12		.43***	.12	.25	.74	
Covariance (men)	.14***	.04	.08	.25		.34***	.09	.20	.59	
Residual variance	.10***	.02	.07	.15		.75***	.08	.54	.88	
*Goodness-of-fit*										
–2 Restricted log likelihood	50.20					100.61				
AIC / BIC	56.20 / 62.22					106.61 / 112.63				
Pseudo *R*^2^—full model (women / men)	65.4% / 0%					11.2% / 30.8%				
Pseudo *R*^2^—invisible support only (women/men)	27.3% / 0%					7% / 30.8%				

*AIC* Akaike Information Criterion; *BIC* Bayesian Information Criterion. Null model for depression: AIC = 96.14; BIC = 104.64; for life purpose: AIC = 152.50; BIC = 161.01.

*p < .05.

**p < .01.

***p < .001.

**Table 6:** Results of MLM of support equity on depression and life purpose (*N* = 31 couples).

Predictor: support equity	Adjustment									
	Depressive symptoms					Life purpose				
	*B*	*SE*	*95% Cl*		*Effect size r*	*B*	*SE*	*95% Cl*		*Effect size r*
			*lower*	*upper*				*lower*	*upper*	
*Fixed effects*										
Intercept	1.02***	.05	.93	1.12		5.52***	.10	5.31	5.73	
Actor support equity	-.35	.20	-.76	.05	.29	.13	.40	-.70	.95	.06
Partner support equity	-.55**	.20	-.95	-.15	.43	.22	.40	-.60	1.05	.10
Infertility duration	.07*	.03	.001	.15	.37	-.20*	.07	-.36	-.05	.46
Gender (-1 women, 1 men)	-.33***	.04	-.42	-.25	.83	.38***	.05	.27	.49	.80
Actor support equity × gender	.26	.20	-.14	.63	.19	-1.01**	.31	-1.36	-.38	.41
Partner support equity × gender	-.01	.19	-.39	.38	.04	-.10	.31	-.73	.52	.04
*[Co-]variances*										
Covariance (women)	.14***	.04	.08	.24		.40***	.11	.23	.68	
Covariance (men)	.11***	.03	.07	.19		.41***	.11	.24	.70	
Residual variance	.12***	.02	.09	.18		.54***	.13	.23	.75	
*Goodness-of-fit*										
–2 Restricted log likelihood	60.48					115.68				
AIC / BIC	66.48 / 72.51					121.68 / 127.70				
Pseudo *R*^2^—full model (women / men)	22.3% / 6.2%					14% / 17.5%				
Pseudo *R*^2^—equity support only (women/men)	17.5% / 2.4%					7% / 0%				

*AIC* Akaike Information Criterion; *BIC* Bayesian Information Criterion. Null model for depression: AIC = 96.14; BIC = 104.64; for life purpose: AIC = 152.50; BIC = 161.01.

*p < .05.

**p < .01.

***p < .001.

### Provided and received support effects

In the case of depressive symptoms, the results of MLM showed significant actor and partner effects of received social support (Table 3). Both effects were negative, indicating a relationship between greater received support and better adjustment (lower depression), which was in line with our hypothesis. In addition, significant interactions between actor and partner effects of received support and gender were observed, demonstrating that this effect differed by gender. Further analysis, using the two-intercept MLM model, found that the actor effect of received support on depressive symptoms was significant only in women, and was negative (*B* = -.67, *SE* = 0.13, *p* < 0.001; men: *B* = 0.06, *SE* = 0.14, *p* = 0.678), whereas the partner effect of received support was significant only in men, and was also negative (*B* = -.50, *SE* = 0.15, *p* = 0.002; women: *B* = 0.06, *SE* = 0.14, *p* = 0.650). These results are in line with our hypothesis.

As for the provided support—depression relationship (Table 4), the actor effect was found to be significant and negative: greater provided support was related to better individual adjustment. Both actor and partner effects were moderated by gender. Further analysis demonstrated that the actor effect of provided support on depressive symptoms was negative and stronger in women (*B* = -.98, *SE* = 0.16, *p* < 0.001) as compared to men (*B* = -.35, *SE* = 0.16, *p* = 0.040), whereas the partner effect of provided support was significant only in men, and was also negative (*B* = -.41, *SE* = 0.18, *p* = 0.031; women: *B* = 0.15, *SE* = 0.15, *p* = 0.315), which is in line with our hypothesis.

In the case of life purpose (Tables 3 and 4), both actor effects of received and provided support were significant, demonstrating that both types of social support had beneficial effects (higher life purpose), supporting our hypothesis. One partner effect was moderated by gender: the effect of provided support had a distinct trend toward significance in women (*B* = 0.58, *SE* = 0.31, *p* = 0.067), and was not significant in men (*B* = -.32, *SE* = 0.28, *p* = 0.271).

### Effects of the discrepancy in support perception

Regarding invisible support effects, there were no main effects of this kind of support on depression or life purpose (Table 5). Gender moderated the effect of partners’ invisible support on depression. The two-intercept MLM model indicated that partners’ invisible support was related to higher depressive symptoms only in women (*B* = 0.62, *SE* = 0.18, *p* = 0.002), and not in men (*B* = -.06, *SE* = 0.29, *p* = 0.835). In addition, gender moderated the effect of actor invisible support on life purpose. Even though this effect was negative and stronger in women (*B* = -.84, *SE* = 0.54, *p* = 0.128) as compared to men (*B* = 0.50, *SE* = 0.41, *p* = 0.240), neither effect was statistically significant.

As for support equity effects (Table 6), a partner effect on depressive symptoms was noted: a greater difference between partner received and provided support (dominance of receipt) correlated with better individual adjustment. The actor support equity effect on life purpose was moderated by gender: it was positive and significant in women (*B* = 1.14, *SE* = 0.52, *p* = 0.037), but not in men (*B* = -.88, *SE* = 0.50, *p* = 0.087).

## Discussion

The aim of this study was to investigate actor and partner effects of support (receipt, provision, invisible, equity) on adjustment in couples undergoing ART treatment.

Received support had a beneficial effect, as it was associated with a lower level of depression and higher life purpose in both partners, supporting social exchange theory [21]. Similar results were obtained by previous studies of couples dealing with cancer [11,38], older couples [39], individuals with arthritis [40], and individuals coping with daily life [41]. Thus, support receipt was not associated with any costs, as had been reported earlier [24,42].

Moreover, gender moderated the relationship between received support and depression. Higher support receipt in women related to lower depressive symptoms in both wives and husbands. No such correlations were found for support receipt in men, consistent with the findings of Martins et al.[10]with regard to perceived support. This supports our hypothesis, indicating that women benefit more from support receipt than do men. Theoretically, these results may be linked to the process of socialization and gender stereotypes: women may be granted more help and support as compared to their male partners, and are more receptive and responsive to supportive behaviors of others as compared to men [43], which allows them to benefit from support. On the other hand, the role of a man is partially defined by instrumentality, success, and power. Thus, their well-being may be higher if their partner confirms she received support. This is also consistent with esteem enhancement theory [14], according to which the possibility of supporting a partner reinforces individual well-being.

As for provided support, its effects were beneficial. Regardless of the type of adjustment (positive *vs*. negative), only actor effects were significant, indicating that higher support provision correlated with higher life purpose and lower depressive symptoms in an individual. These findings are consistent with esteem enhancement theory, such that supporting others is an antecedent of increased well-being [14], and earlier studies on the effect of support provision in couples [11–13,44].

The effects of provided support on depression were gender-moderated. A more detailed analysis demonstrated the actor effect to be stronger in women as compared to men. A more beneficial effect of provided support was therefore observed in women. This finding is consistent with the female stereotype: supporting others and giving more attention to supportive interactions [43]. Furthermore, the partner effect was significant only in men, indicating that higher provided support reported by women related to lower depressive symptoms in men. In other words, more supportive female partners positively correlated with higher well-being of their male partners. Several hypotheses might explain this dependence. First, based on integrated resource theories [45], it is possible that a more supportive wife has more resources in general (in accordance with resource caravans and resource gain cycles), resulting in better coping with the challenges of infertility and not burdening the partner and the relationship. Consequently, the husband’s well-being is higher. Second, a supportive wife also has higher well-being. The mood of the wife may be the mediating variable between the support provision of the wife and the mood of the husband, as the literature supports the thesis that the mood of one spouse affects the mood of the other spouse [44,46]. Third, received support might be a mediator or a moderator of the abovementioned relation. Supposedly, higher support provision from the wife results in higher visible or invisible support receipt of the husband. Regardless of the degree of visibility, supportive female partners have a highly beneficial effect.

The analysis of the discrepancy between support receipt and provision in each partner only partially supported social exchange theory, that is, the advantages of support receipt. For women only, dominance of received support was related to benefits to themselves, that is, higher life purpose. That relationship with depression was, in turn, characterized by the partner effect, indicating that the other partner also benefited from the partner’s predominance of support receipt. Of note, there were no gender differences in received and provided social support. In addition, both supports were at similar levels in the entire sample.

Regarding invisible support, its hypothesized relations to greater well-being and better stress resistance were not supported. Moreover, when gender differences were considered, invisible support even tended to “backfire”. Higher actor’s invisible support was associated with lower actor’s life purpose. This tendency was stronger among women as compared to men. In addition, higher invisible support reported by men was related to higher depressive symptoms in women. Thus, our hypothesis concerning greater benefit from invisible support in affected women was not supported. These findings are consistent with conflicting results in the literature on the effects of invisible support. Regardless of the fact that numerous authors have confirmed its beneficial effect [23–25], there are also studies that contradict that theory [47]. Based on the presented findings, it can be concluded that both partners, especially women, anticipated visible support. Visible support favors conformation of support receipt, expressing gratitude, and reciprocation, which might be welcome in the context of coping with infertility. On the other hand, the result may in fact mirror the abovementioned differences in perceiving support interactions by women and men, which is determined by their social roles.

In a practical sense, it seems prudent to conduct interventions aimed at educating partners about effective support provision, adjusted to their mutual needs in terms of demand and quality, and paying attention to supportive interactions. Another practical implication might be the need for an intervention on support perception (provision and receipt) in men, for them to benefit more from support. Finally, since women are at risk for maladjustment in these settings, attention of clinicians ought to be directed toward that gender group. Furthermore, a consistent amount of care throughout infertility treatment is necessary to avoid deteriorating care over time, which may be one of the main causes of a correlation between treatment duration and poorer adjustment.

These findings have important theoretical and practical implications. Nevertheless, the study was not without limitations. First, generalization of the study results is limited due to the relatively low response rate. Second, the sample size can also be considered to be small, particularly in terms of the number of couples. Still, adequate power in the test of nonindependence [34] and the unbiased estimates of the fixed effects parameters could be obtained for this sample [35–37]. Access to a larger and more diversified population was restricted by the internal regulations of many clinics, most of which were private, which relates to the lack of bioethical laws in Poland. Thus, only patients of the public infertility center (with state-funded *in vitro* treatment) were recruited. Moreover, the partner clinic had little patient rotation. Furthermore, the study was cross-sectional, which does not allow us to draw cause-effect conclusions about the investigated relationships. Future studies will benefit from a larger sample size and longitudinal design. Despite these limitations, these findings highlight the distinct effects of visible support receipt and provision, and invisible support on both positive and negative aspects of adjustment in infertile couples, and hopefully will inspire future research in this area.
